# Evaluation of Risk Factors Associated with Reinfarction: A Multicenter Observational Study

**DOI:** 10.7759/cureus.6063

**Published:** 2019-11-03

**Authors:** Kheraj Mal, Inayatullah D Awan, Faizan Shaukat

**Affiliations:** 1 Cardiology, National Institute of Cardiovascular System, Sukkur, PAK; 2 Psychiatry, Ghulam Muhammad Mahar Medical College, Sukkur, PAK; 3 Internal Medicine, Jinnah Postgraduate Medical Centre, Karachi, PAK

**Keywords:** mi, myocardial infarction, reinfarction, risk factors

## Abstract

Introduction: Reinfarction after incidence of myocardial infarction is a serious complication and is responsible for high mortality. Various factors are responsible for reinfarction including smoking, prior procedures or surgeries, and use of medications such as aspirin, β-blocker, and angiotensin-converting enzyme Iihibitor or angiotensin receptor blockers.

Material and Methods: This prospective study was conducted with 243 participants. Participants were divided into two groups: patients who had a reinfarction during hospital and patients who did not.

Results: There were 142 (58.4%) men and 101 (41.6%) women in the study. A total of 17 (6.9%) patients had reinfarction. Age (68.4±10.9 vs. 64.4±11.8; 0.001), diabetes (47.05% vs. 22.12%; 0.02), and history of myocardial infarction (29.5% vs. 11.4%; 0.02) were identified as risk factors for reinfarction

Conclusion: Our study reports that certain parameters such as age, obesity, diabetes mellitus,, and history of myocardial infarction can be used to assess the risk of reinfarction among these patients.

## Introduction

Reinfarction after ST-elevation myocardial infarction (MI) is a very serious occurrence, and it leads to high mortality among patients. Recurrent MI or reinfarction is defined as recurrence of clinical signs and symptoms of ischemia in patients with previously diagnosed MI, with accompanying electrocardiographic changes and raised serum biomarker levels consistent with myocardial necrosis [1]. Reinfarction is one of the major causes of morbidity and mortality in patients with known cardiac disease.

Incidence of reinfarction is reported to be significantly associated with factors including smoking, prior procedures or surgeries such as percutaneous coronary intervention (PCI) or bypass graft, and use of medications such as aspirin, β-blocker, and angiotensin-converting enzyme inhibitor or angiotensin receptor blockers [2]. Multiple studies suggest that MI patients with a history of stroke are more prone to reinfarction. The use of medication to regulate cholesterol levels has been shown to reduce the incidence of reinfarction [2,3].

One important factor that provides beneficial effect after MI is the use of warfarin therapy. It has been shown to reduce the risk of recurrent infarction and mortality in patients [4]. Other factors reported in the literature that significantly contribute to reinfarction are preoperative hypertension, intraoperative hypotension, and surgeries of long duration [4,5].

Recurrent MI is still a very common complication faced by the patients with a history of MI, despite the progress in new therapeutic modalities and introduction of more efficient pharmacotherapy and stents [2]. This study aimed to evaluate the risk factors associated with myocardial reinfarction in patients with MI in a multicenter study.

## Materials and methods

This was a prospective study conducted from 1 January 2018 to 30 December 2018 in cardiology unit of tertiary care hospital in Sukkur. A total of 281 participants were admitted to cardiology unit with diagnosis of MI during the study period. A total of 29 participants expired and 19 participants refused to participate in this study. A total of 243 participants were enrolled in this study.

Participants were divided into two groups: patients who had a reinfarction during hospital and patients who did not. Reinfarction was defined as a renewed increase in cardiac enzymes, with or without chest pain and/or electrocardiographic changes. For the diagnosis, cardiac enzymes were measured at least once every 24 hours. We analyzed the demographic characteristics, coronary history, and coronary risk factors.

Data were analyzed using SPSS Version 21.0 (Armonk, NY: IBM Corp.). Mean and standard deviation were calculated for continuous variables such as average, while frequencies and percentages were calculated for categorical variables including gender and risk factors.

## Results

There were a total of 243 participants in study. The mean age was 64.4±13.13 years. There were 142 (58.4%) men and 101 (41.6%) women in the study. A total of 17 (6.9%) patients had reinfarction. Age (68.4±10.9 vs. 64.4±11.8; 0.001), diabetes (47.05% vs. 22.12%; 0.02), and history of MI (29.5% vs. 11.4%; 0.02) were identified as risk factors for reinfarction. Remaining risk factor and histories for reinfarction and non-reinfarction groups are given in Table 1.

**Table 1:** Comparsion of Risk Factors BMI, body mass index; MI, myocardial infarction

Characteristics	Reinfarction MI (n=17)	Non-reinfarction MI (n=226)	P value
Age	68.4±10.9	64.4±11.8	0.001
Smoking	6 (35.29%)	72 (31.85%)	0.7
Hypertension	9 (52.94%)	110 (48.67%)	0.73
Hypercholesterolemia	6 (35.295)	74 (32.74%)	0.82
Diabetes	8 (47.05%)	50 (22.12%)	0.02
Obese (BMI greater than 30 mg/kg^2^)	5 (29.4%)	61 (26.9%)	0.04
Previous MI	5 (29.5%)	25 (11.4%)	0.02
Previous angina	5 (29.4%)	40 (17.6%)	0.23

## Discussion

Recurrent infarction after an acute MI or primary PCI is one of the most important prognostic factors and a cause of high mortality among patients with cardiac diseases [6]. Our study reported incidence of reinfarction to be as high as 6.9%, which is comparable to other studies. In a large-scale, multinational HORIZONS-AMI (Harmonizing Outcomes with Revascularization and Stents in Acute Myocardial Infarction) trial conducted in 2014 with an enrollment of over 3,000 patients, the incidence of reinfarction was reported to be as 1.8% at one month, 4.0% at one year, and 6.9% at three years [2]. In Thrombus Aspiration during Percutaneous coronary intervention in Acute myocardial infarction Study (TAPAS), a reinfarction rate of 3.4% was reported [7].

In other large-scale studies, the incidence of reinfarction after primary PCI was reported to be between 2% and 4.5% within one month and of 6% within one year after primary PCI [8-11]. These findings are in concordance with our results.

In two large-scale German registries known as Maximal Individual Therapy in Acute Myocardial Infarction (MITRA) and the Myocardial Infarction Registry (MIR), with over 22,613 patients with acute MI, the incidence of reinfarction was found to be 4.7% [12].

One of the most dreaded complications with PCI with stents is formation of stent thrombosis, and it has a significant calamitous effect on the outcome for the patient. In many studies, stent thrombosis was responsible for increased risk of reinfarction in patients after intervention with stents [2,6-11]. However, no evidence was found between the type of stent used and incidence of reinfarction [13].

In our study, association of occurrence of reinfarction with a series of variables including age, smoking, hypertension, hypercholesterolemia, diabetes, obesity, and history of MI or angina was explored. Older age (p=0.001), diabetes mellitus (p=0.02), obesity (p=0.04), and history of MI (p=0.02) were significantly associated with the incidence of reinfarction.

Age and history of MI have also been reported to be significantly associated with reinfarction according to the MITRA-MIR study [12]. Similarly, according to the PRIMVAC registry, after multivariate analysis age, obesity, history of MI, and Q wave on electrocardiogram were independently associated with reinfarction after acute MI [6].

These findings are in contrast to those reported by Birnbaum et al, who did not find any significant association between a history of MI and reinfarction [14]. However, there is to a certain extent a consistency in the literature with respect to the predictive role of age and history of MI with occurrence of reinfarction. Those with multivessel coronary disease are more at risk of developing reinfarction after acute MI [2].

In our study, diabetes mellitus was significantly correlated with reinfarction. This is supported by the study conducted by Birnbaum et al [14]. In contrast, PRIMVAC and MITRA-MIR studies did not find any significant correlation between reinfarction after acute MI and diabetes mellitus. However, they reported a higher number of diabetics in the group with reinfarction [6,12].

In our study, no significant association was found between gender and reinfarction. This is in contrast to the MITRA-MIR study, which found a significant association between female gender and incidence of reinfarction [12]. In some studies, reduced left ventricular ejection fraction, coronary stenosis of greater than 30%, presence of coronary dissection, and stent thrombosis significantly correlated with recurrent MI [15]. More research is needed to explore these variables. 

A CHA2DS2-VASc score is often used, and it is a good predictor for the risk assessment of adverse complications. It includes variables such as hypertension, diabetes mellitus, smoking, and heart failure in individuals with acute MI [16]. The early detection and aggressive approach towards the management of those patients with an increased risk for reinfarction after MI can reduce the incidence of reinfarction and a subsequent decrease in mortality and morbidity [17,18].

To summarize, our study reports that certain parameters such as age, obesity, diabetes mellitus, and history of MI can be used to assess the risk of reinfarction among these patients.

## Conclusions

Age, diabetes, and history of MI were identified as risk factors for reinfarction. Efforts should be made to identify risk factors in reinfarction. In conclusion, improvements in types of stents used and advancement in pharmacological interventions including antiplatelet with lesser bleeding risk can help reduce the risk of reinfarction and other complications related to MI.

## References

[1] Cannon CP, Brindis RG, Chaitman BR (2013). 2013 ACCF/AHA key data elements and definitions for measuring the clinical management and outcomes of patients with acute coronary syndromes and coronary artery disease: a report of the American College of Cardiology Foundation/American Heart Association Task Force on Clinical Data Standards (Writing Committee to Develop Acute Coronary Syndromes and Coronary Artery Disease Clinical Data Standards). J Am Coll Cardiol.

[2] Stone SG, Serrao GW, Mehran R (2014). Incidence, predictors, and implications of reinfarction after primary percutaneous coronary intervention in ST-segment-elevation myocardial infarction: the Harmonizing Outcomes with Revascularization and Stents in Acute Myocardial Infarction Trial. Circ Cardiovasc Interv.

[3] Tian L, Yang Y, Zhu J (2016). Impact of previous stroke on short-term myocardial reinfarction in patients with acute ST segment elevation myocardial infarction: an observational multicenter study. Medicine.

[4] Le May MR, Acharya S, Wells GA (2015). Prophylactic warfarin therapy after primary percutaneous coronary intervention for anterior ST-segment elevation myocardial infarction. JACC Cardiovasc Interv.

[5] Norris RM, Barnaby PF, Brandt PW (1984). Prognosis after recovery from first acute myocardial infarction: determinants of reinfarction and sudden death. Am J Cardiol.

[6] Ahumada M, Cabadés A, Valencia J (2005). Reinfarction as a complication of acute myocardial infarction. PRIMVAC Registry data. Rev Esp Cardiol.

[7] Fokkema ML, Van Der Vleuten PA, Vlaar PJ, Svilaas T, Zijlstra F (2009). Incidence, predictors, and outcome of reinfarction and stent thrombosis within one year after primary percutaneous coronary intervention for ST‐elevation myocardial infarction. Catheter Cardiovasc Interv.

[8] De Luca G, Ernst N, van't Hof AW (2006). Predictors and clinical implications of early reinfarction after primary angioplasty for ST-segment elevation myocardial infarction. Am Heart J.

[9] Simoons M, Ellis S (1997). A clinical trial comparing primary coronary angioplasty with tissue plasminogen activator for acute myocardial infarction. N Engl J Med.

[10] Smit JJ, van’t Hof AW, de Boer MJ (2006). Incidence and predictors of subacute thrombosis in patients undergoing primary angioplasty for an acute myocardial infarction. Thromb Haemost.

[11] Gopalakrishnan M, Lotfi AS (2018). Stent thrombosis. Semin Thromb Hemost.

[12] DÖnges K, Schiele R, Gitt A (2001). Incidence, determinants, and clinical course of reinfarction in-hospital after index acute myocardial infarction (results from the pooled data of the maximal individual therapy in acute myocardial infarction [MITRA], and the myocardial infarction registry [MIR]). Am J Cardiol.

[13] Mehran R, Brodie B, Cox DA (2008). The Harmonizing Outcomes with RevasculariZatiON and Stents in Acute Myocardial Infarction (HORIZONS-AMI) trial: study design and rationale. Am Heart J.

[14] Birnbaum Y, Herz I, Sclarovsky S (1998). Admission clinical and electrocardiographic characteristics predicting an increased risk for early reinfarction after thrombolytic therapy. Am Heart J.

[15] Kernis SJ, Harjai KJ, Stone GW (2003). The incidence, predictors, and outcomes of early reinfarction after primary angioplasty for acute myocardial infarction. J Am Coll Cardiol.

[16] Bozbay M, Uyarel H, Cicek G (2017). CHA2DS2-VASc score predicts in-hospital and long-term clinical outcomes in patients with ST-segment elevation myocardial infarction who were undergoing primary percutaneous coronary intervention. Clin Appl Thromb Hemost.

[17] Rousan TA, Thadani U (2017). Stent choice in cardiogenic shock complicating acute myocardial infarction likely does not affect mortality or reinfarction. BMJ Evid Based Med.

[18] Castro-Dominguez Y, Dharmarajan K, McNamara RL Predicting death after acute myocardial infarction. Trends Cardiovasc Med.

